# Supplementary material for: Characterisation of oxygen defects and nitrogen impurities in TiO2 photocatalysts using variable-temperature X-ray powder diffraction
Source: Nat Commun. 2021 Jan 28;12:661. doi: 10.1038/s41467-021-20977-z (PMC7844033; doi:10.1038/s41467-021-20977-z)
Supplement: Supplementary file 1 — Supplementary Information [file 41467_2021_20977_MOESM1_ESM.pdf]

Supplementary information

Thermal expansion of P25 and N-P25

With respect to the refinement, certain instrumental errors were corrected by the structural refinement of a silicon-standard pattern. The lattice parameter of silicon is known to very high accuracy, 5.431020511 Å [2]. The ten-significant-figure value was fixed, and the wavelength and angular zero-error allowed to refine, yielding a wavelength accurate to six decimal places when expressed in angstroms 0.824875(1) Å, and a zero-error of +0.00467(5). The six- and three-decimal place values were used in all subsequent refinements.

The calibration of the sample temperature was conducted prior to investigation by measuring the thermal expansion of platinum metal powder at room temperature and at 100, 200, 300, 400 and 500°C. Patterns were rebinned to 8 mdeg., then the lattice parameters determined by Pawley refinement (average R_{wp} = 12.53). These values had a standard uncertainty in the order of 1×10^{-5} Å. The experimental lattice parameters were compared to the theoretical values (seven-term polynomial[1]) to yield the real sample temperature, which had associated standard uncertainties below 2°C. The relationship was fitted well by a linear function (m = 0.8979260, c = 0.8694716; R = 0.9999), with the calibrated temperatures found to be an average of 10% below the input temperature. The remaining input temperatures (i.e. for 50°C, 150°C, 250°C, 350°C and 450°C) were calculated by linear interpolation between the two closest calibration points (Table S2, Figure S10). These interpolated values exhibited slightly higher errors, but the greatest still being low (±2°C for 150°C calibrated to 134°C). These errors are carried through the first-derivative calculations.

Error analysis

For the variable temperature analysis of P0 and P3, lattice parameters were refined by Pawley refinement. The relative standard uncertainties are typically of the order 10^{-5} or $\pm1\times10^{-5}$ Å. This is equivalent to a relative error of the order 1×10^{-5} or 0.001%. The effect of using traditional peak shape parameters or size/strain analysis by fundamental parameters yielded very similar errors. Rebinning the data also had negligible effect.

Though this relative uncertainty may seem insignificant, the propagation of error associated with calculation of thermal expansion coefficients is significant. The value Δa is much smaller than the absolute lattice parameters; hence the relative uncertainty is much higher. The resulting thermal expansion coefficients typically have a relative uncertainty of ~10%. This is a particular problem at low temperature when the thermal expansion is very low. No methodological errors have been identified.

Input temperature / °C	Measured a / Å	SD / Å	Δa / Å	SD / Å	$\Delta a/a_0$	SD	Calibrated temperature / °C	SD / °C
22.5	3.923798	0.000008	0	0.0000113	0		22.5	
100	3.926209	0.00001	0.002411	0.0000128	0.0006145	0.000003	88.1	1.92
200	3.929578	0.000011	0.00578	0.0000136	0.0014731	0.000003	180.7	1.07
300	3.93297	0.000011	0.009172	0.0000136	0.0023375	0.000003	271.7	0.81
400	3.936406	0.000011	0.012608	0.0000136	0.0032132	0.000003	361.88	0.68
500	3.93986	0.000012	0.016062	0.0000144	0.0040935	0.000004	450.57	0.65

Table S1. Temperature calibration and error propagation for variable-temperature synchrotron X-ray powder diffraction on I11, DLS. Fits to a linear function: m = 0.8979260, c = 0.8694716; R = 0.9999.

Input temperature / °C	Temp. from T below (x_i-x_1) / °C	T interval between points (x_2-x_1) / °C	Fraction between points	Experimental T interval (y_2-y_1) / °C	SD / °C	Temp. above lower point / °C	SD / °C	Interpolated temperature / °C	SD / °C
50	27.5	77.5	0.355	65.60	1.92	23.28	0.68	45.78	0.68
150	50	100	0.500	92.60	2.20	46.30	1.10	134.40	2.21
250	50	100	0.500	91.01	1.34	45.50	0.67	226.20	1.26
350	50	100	0.500	90.18	1.06	45.09	0.53	316.79	0.97
450	50	100	0.500	88.69	0.94	44.34	0.47	406.23	0.83

Table S2. Interpolated temperatures and error propagation by linear interpolated between two closest experimental values.

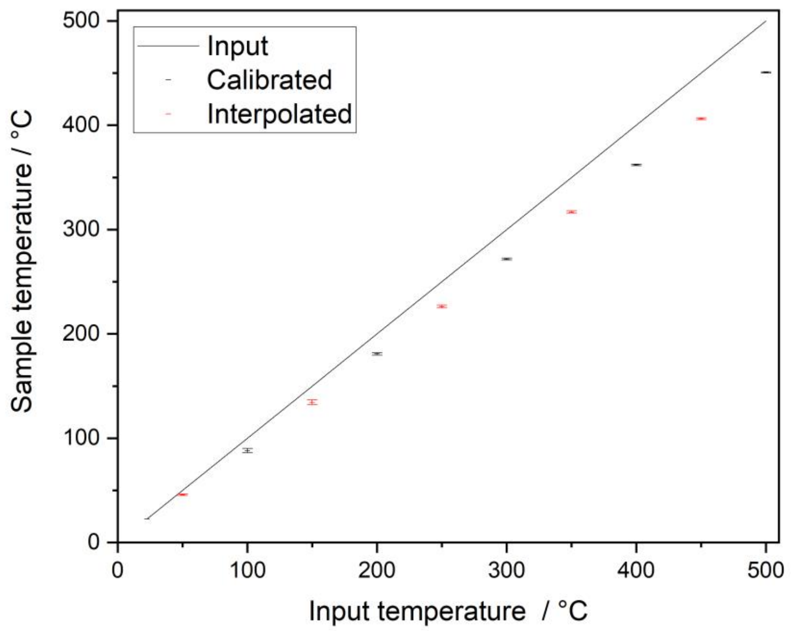

Figure S1. Calibration of input temperature (hot air blower) to sample temperature by using the well-known thermal expansion of platinum metal. Experimental data in black; interpolated values in red.

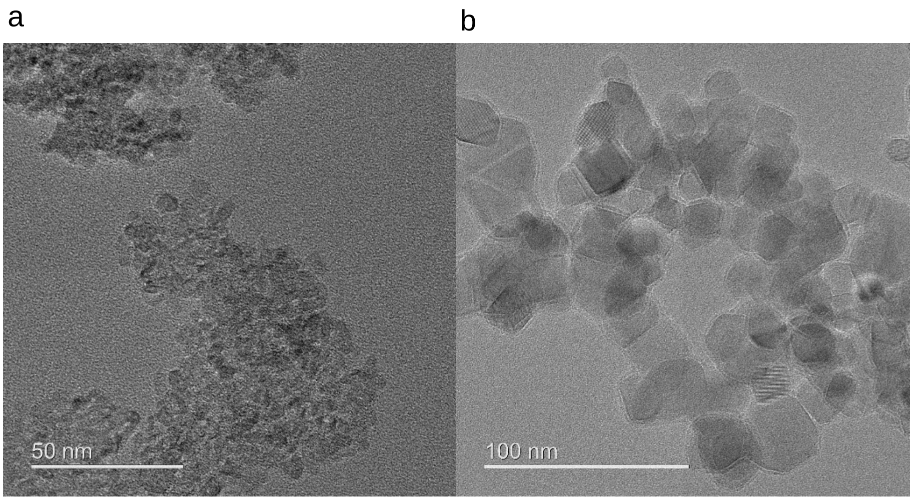

Figure S2. TEM micrographs showing typical morphology of (a) A3, and (b) P3.

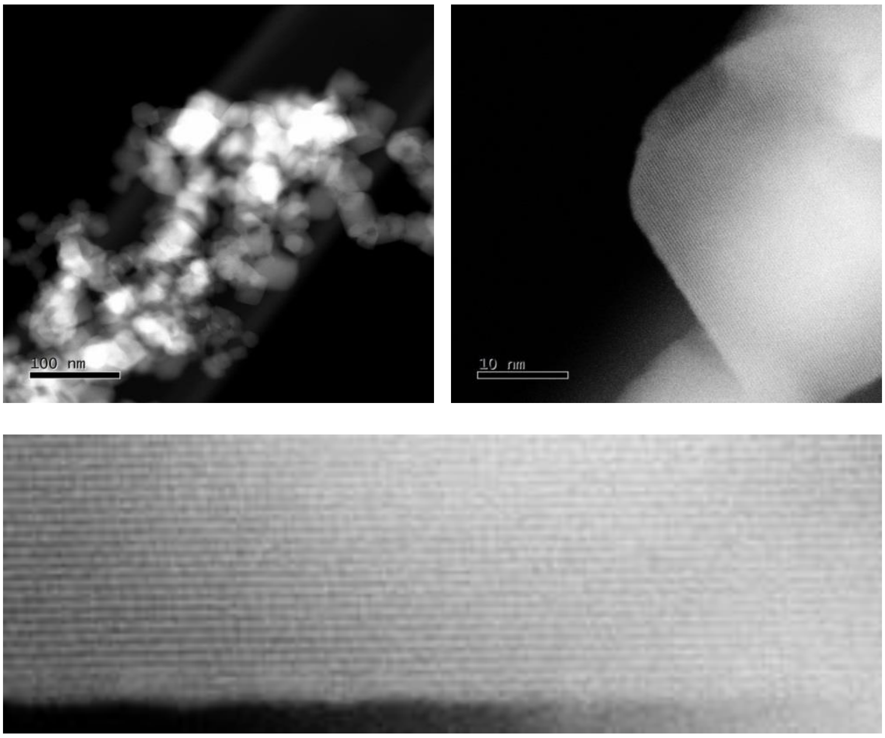

Figure S3. HAADF-STEM micrographs of P3 show the typical lattice spacing of (101) anatase TiO_2 in the bulk structure (0.35 ± 0.02 nm). However, at the surface a few atomic layers exhibit distorted lattices.

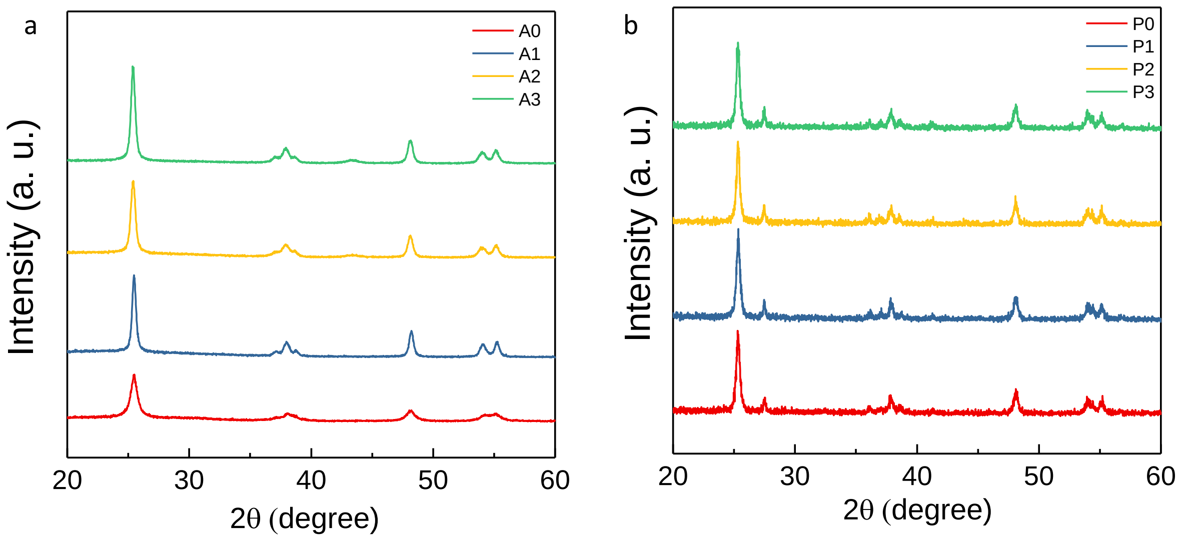

Figure S4. Laboratory-source XRD patterns of N-doped TiO_2 materials treated with ammonia at different temperatures (a) pure phase anatase and, (b) P25.

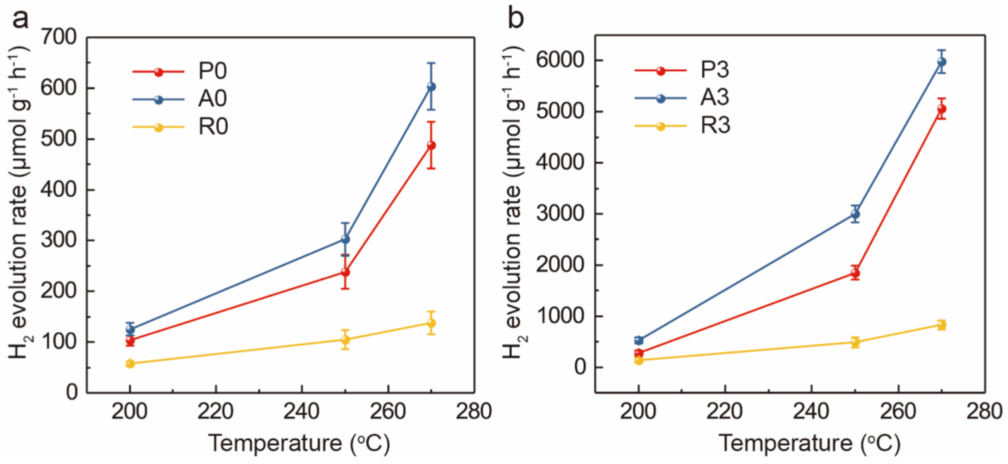

Figure S5. The temperature dependences of photocatalytic water splitting activity over (a) pristine, and (b) N-doped anatase, rutile, and P25 samples. Since the pristine anatase, rutile and P25 samples show negligible absorption in the visible light range, a Xe lamp, which contains UV light from 300 nm and also visible light, was used as the light source for the photocatalytic performance tests. The other conditions were all maintained the same. Error bars indicate the standard deviation.

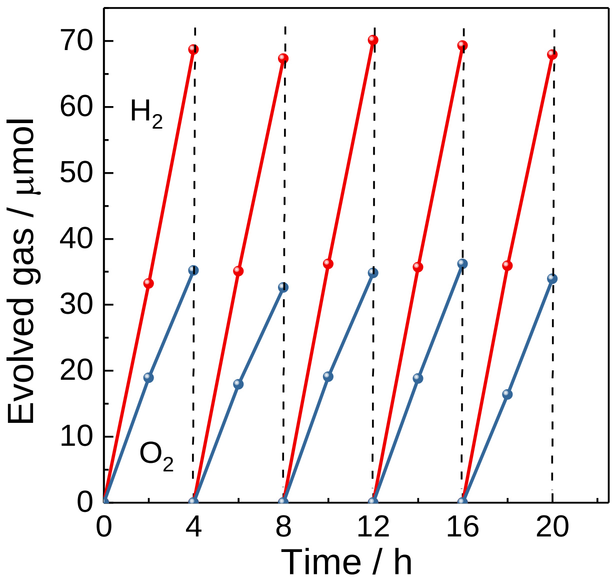

Figure S6. 5-cycle photocatalytic overall water splitting tests of P3. H_2 and O_2 evolved in a stoichiometric 2:1 ratio in each cycle at 270°C.

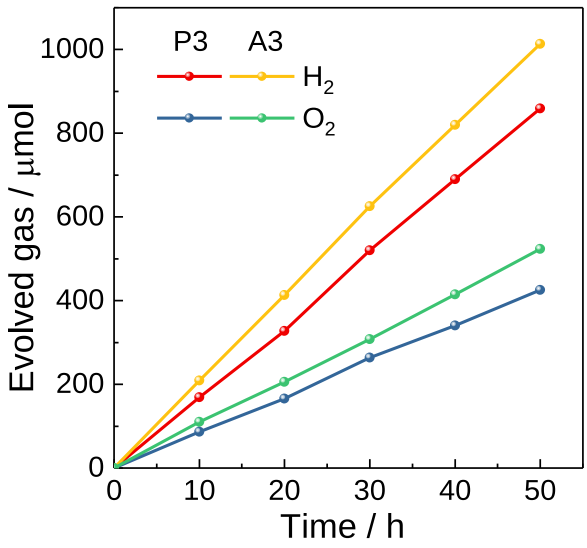

Figure S7. Stable stoichiometric evolution of H_2 and O_2 from photocatalytic overall water splitting reaction over A3 and P3 catalysts without any sacrificial reagents at 270°C over 50 h.

We have carried out a 5-cycle photocatalytic water splitting reaction test in the same batch, of which the results are shown in the revised manuscript. As can be seen in the Fig. S5, hydrogen and oxygen are produced stoichiometrically in 2:1 ratio, which indicates that this reaction is photocatalytic overall water splitting reaction. Also, there was no sign of nitrogen formation from the nitride was observed in the careful GC analysis. We have also carried out a 50-hour long durability test as shown in Fig. S6 in the revised manuscript, which also shows the stable hydrogen and oxygen production in 2:1 ratio within experimental error for over 50 hours with absolutely no N_2 evolution. It is noted that N^{3-} electro-oxidation to N_2 despite its high concentration still requires significantly higher applied potential than that of OH^- to take place and the nitride oxidation can only be favourable in molten medium in the total absence of water[1]. Thus, the possibility that the doped N anions act as hole-scavengers in the presence of water is not likely to happen.

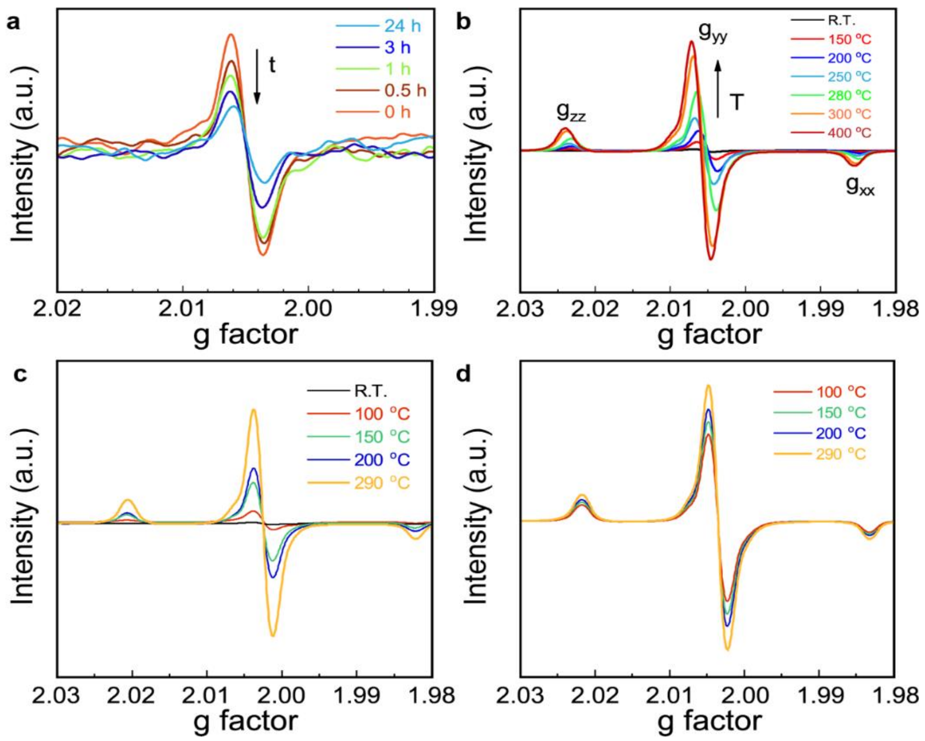

Figure S8. EPR spectra of P1 (a) at times following thermal treatment. Deactivated P1 re-calcined at various temperatures in: (b) N_2, (c) liquid water, and (d) water vapour. Reproduced from reference [4].

P1 treated with 550°C in NH_3 was prepared and exposed to air for different time ranges. Interestingly, after the sample was exposed to air at ambient conditions for 1.5 hours, 40% of the EPR signal, that is indicative of the presence of surface oxygen vacancies, gradually disappeared and after 24 hours, only 23% of the original signal remained. This is attributed to the fact that oxygen sources in the air (i.e. O_2 and H2O), when in contact with the particle surface, may gradually replenish the surface oxygen vacancies and redistribute the electrons, approaching the electronic configuration of pristine TiO_2. This could explain the fact that N-doped TiO_2 or hydrogenated TiO_2 do not necessary show good photocatalytic water splitting activity under visible light illumination in air even though the remaining oxygen defects in bulk can exert strong visible light absorption. However, we also noticed that re-calcining the N-doped TiO_2 in a N_2 atmosphere at elevated temperatures, the EPR signals of these materials re-emerge and become even larger, implying that more surface oxygen vacancies are regenerated at elevated temperatures (Figure S8a). Thus, the surface oxygen vacancies formed in N-doped TiO_2 are vulnerable to oxygen sources at room temperature, but at elevated temperatures (Figure S8b and c), the faster subsequent reactions can regenerate them to sustain the surface photocatalytic processes. Relevant analysis from directly reproduced from reference[4].

Oxygen vacancies can trap unpaired electrons from the semi-conductive oxide, which is detectable by electron paramagnetic resonance (EPR). Thus, EPR measurements were carried out after P1 was freshly prepared and exposed to air for different time ranges (Figure S8a). Interestingly, after the sample was exposed to air at ambient conditions for 1.5 hours, 40% of the EPR signal, that is indicative of the presence of oxygen vacancies, gradually disappeared and after 24 hours, only 23% of the original signal remained (Figure S8a). This is attributed to the fact that oxygen sources in the air (i.e. O_2 and H_2O), when in contact with the particle surface, may gradually replenish the oxygen vacancies and redistribute the electrons, approaching that of pristine TiO_2. This could explain the fact that N-doped TiO_2 or hydrogenated TiO_2 do not necessary show good photocatalytic water splitting activity at room

temperature under visible light illumination in air even though the remaining oxygen defects in bulk (require to diffuse to surface but the rate is very slow) can exert strong visible light absorption.

However, we also noticed that after re-calcining the N-doped TiO_2 in a N_2 atmosphere at elevated temperatures, the EPR signals of these materials re-emerge and become even larger, implying that more oxygen vacancies are regenerated at elevated temperatures (Figure S8b)). Thus, the surface oxygen vacancies formed in N-doped TiO_2 are vulnerable to oxygen sources at room temperature, but at elevated temperatures, the faster subsequent reactions can regenerate them to sustain the surface photocatalytic processes. According to the post-mortem analysis measured by EPR, it is apparent that the EPR signals increase in a similar way in the conditions with liquid water and water vapour as that in pure N_2 (Figure S8c,d), which suggests that the oxygen vacancies can still be regenerated at elevated temperatures under the photocatalytic water splitting conditions. However, we believe that the presence of water may still affect the adsorptive properties of surface oxygen vacancies in the samples as well as their relative stability of the subsurface vacancies.

Although we have not yet able to address the effect of water on the oxygen vacancy or sub oxygen vacancy during the catalysis in this work, previous publications[5], and their corresponding discussions may give readers some information about their potential impacts in this area.

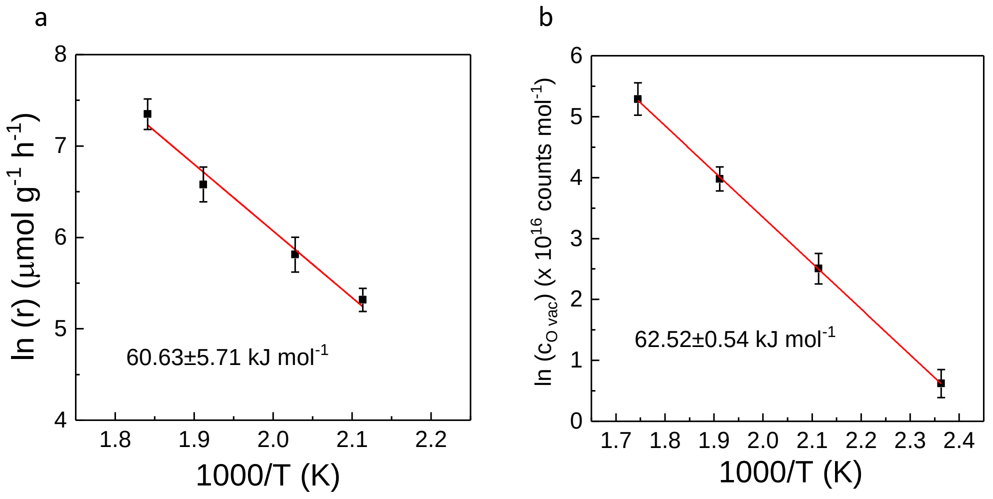

Figure S9. Calculated energy barriers for P1: (a) photocatalytic water splitting reaction calculated using activity against temperature. (b) Oxygen vacancy formation calculated using EPR data against temperature.

DFT Calculations

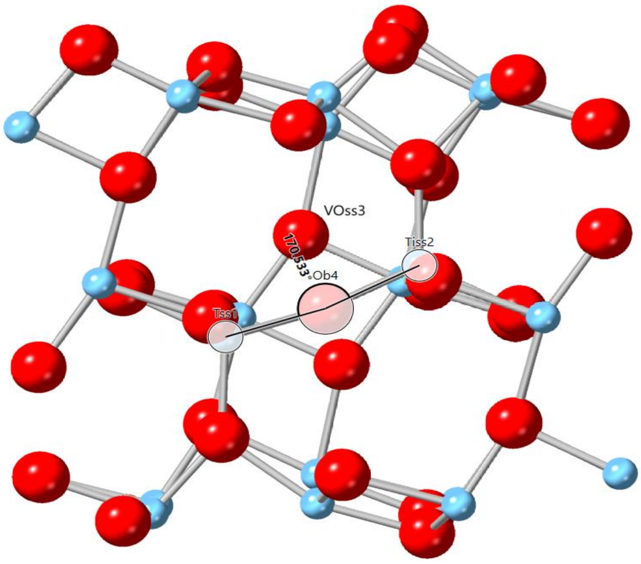

Figure S10. Oxygen vacancy structure V_{Oss3} showing the movement of bulk oxygen atom O_{b4} towards the vacancy, and associated Ti_{ss1}-O_{b4}-Ti_{ss2} bond angle; oxygen (red), titannium (blue).

For further description of structural reorganisation of oxygen defected anatase, the removal of the Oss3 oxygen results in an incomplete four-membered ring. The remaining Ti_{ss1}-O_{b4}-Ti_{ss2} unit distorts from a near right angle (97.7°) to nearly linear (170.5°) observed by SXPD, as the remaining oxygen atom moves significantly towards the vacancy/surface and is effectively no longer associated with the deeper tri-layer (Ti_{b2}-O_{int}: 2.134 Å to 3.695 Å) (Fig. S10). This new oxygen site can be described as pseudo-interstitial. As discussed above, if no structural reorganisation is considered, an oxygen vacancy can be assumed to yield three under-coordinated square pyramidal titanium sites: two with equatorial vacancies and one with an axial vacancy. However, considering the interstitial oxygen observed here, an additional under-coordinated titanium site is shown to form in the bulk tri-layer, resulting in even greater distortion than expected. The differing geometries of the four under-coordinated titanium atoms will evidently influence their orbital configurations and their electron trapping capabilities. It can be shown by crystal field stabilisation theory that the in-plane square pyramidal geometry of the surface species causes partial loss of degeneracy in the titanium lowest unoccupied orbitals, t_{2g}. However, the degeneracy that remains is not conferring maximum stabilisation to d^1 species. On the other hand, the out-of-plane square pyramidal geometry of the sub-surface species causes the complete loss of degeneracy in the lowest unoccupied orbital, and hence maximum stabilisation of d^1 species. Hence, it is proposed that the excess electrons that result from the abstraction of neutral oxygen atoms are localised on the sub-surface under-coordinated titanium atoms Tiss1 and Tiss2. It is emphasised this is due to the interstitial co-linear oxygen site.

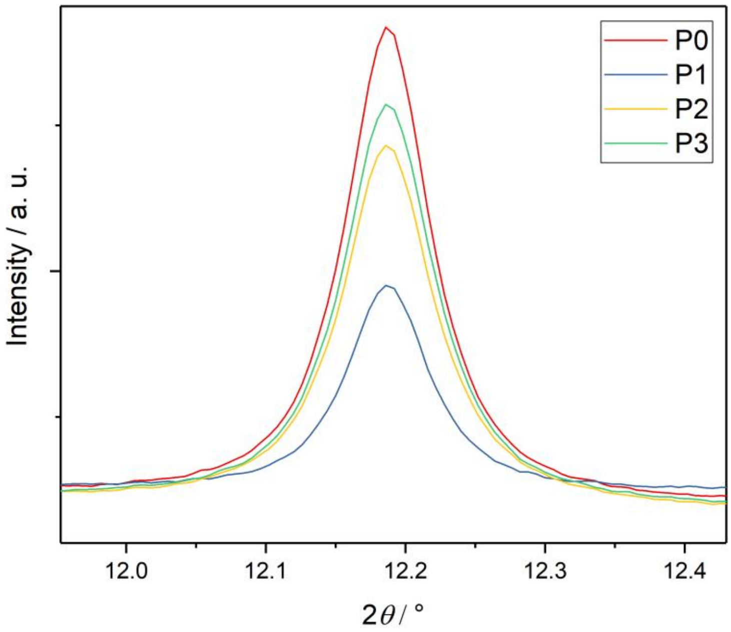

Figure S11. SXPD patterns of anatase samples P0-P3, enlarged view of the peak associated with the (101) reflection.

Titanium oxynitride phase analysis

Refinement

Rietveld refinement was conducted on the room temperature scan of A5 between 4.000°–78.216° in 2θ. The instrumental angular resolution of 0.006° in 2θ was used. Initially an anatase phase ($I4_1/amd$), a titanium nitride ($Fm\bar{3}m$) phase, and 20 background parameters were used. The axial divergence and sample absorption were initially refined then fixed. For the anatase phase, the following parameters were refined: lattice parameters *a* and *c*, oxygen site *z* co-ordinate, titanium and oxygen site thermal parameters, pseudo-Voigt peak shape. For the titanium nitride phase, the following parameters were refined: lattice parameters *a*, titanium and nitrogen site thermal parameters, pseudo-Voigt peak shape. The modelling the cubic phase as titanium nitride with fixed occupancies of unity yielded an unsatisfactory fit, in particular for the reflections (111) (*d* = 2.415 Å) and (200) (*d* = 2.092 Å).

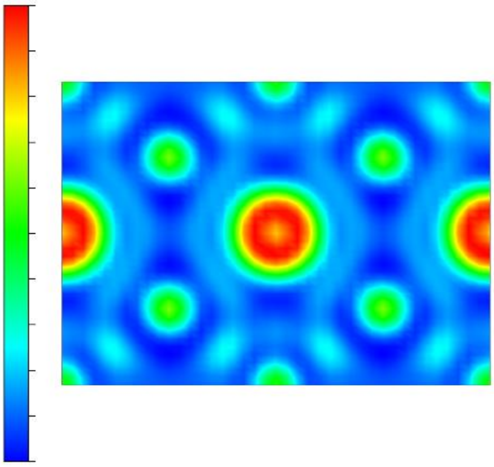

Figure S12. Fourier difference electron density map for unity occupancy of Ti^{3+} and N, which shows very large unmodelled electron density at the nitrogen site (0, 0, 0.5) and the tetrahedral hole (0.25, 0.25, 0.25); titanium site is observed in the F_{diff} transform due to imperfect fitting. Plane shown is at one unit-cell length *a* from the origin along (011).

The following structure factors and phase information were output: $|F_{obs}|$, $|F_{calc}|$, A (= $A_{01} \times B_{11}$), B (= $B_{01} \times A_{11}$). By applying the phase information from the Rietveld refinement to the observed structure factors, 'observed' electron density maps were able to be synthesised. From this set of structure factors, a Fourier difference map can also be synthesised by subtracting the calculated electron density from the observed electron density. In order to analyse the temperature dependence on the structure, a batch Rietveld refinement was carried out. As before, the refined values were automatically used in the following refinement.

Table S3. Structural information for Rietveld refinements of A5 using an anatase and titanium nitride phase.

	structural model	$Ti^{3+}N$	$Ti^{3+}N^{3-}(O^{2-})$	$Ti^{3+}N^{3-}(O^{2-})O_{int}$
residuals	R_{wp} / %	3.3797	3.3871	3.34806
	R_{exp} / %	1.7067	1.7065	1.7062
	GoF	1.9803	1.9848	1.9622
anatase	space group		$I4_1/amd$	
	lpa / Å	3.78517(1)	3.78517(1)	3.7851(1)
	lpc / Å	9.51065(6)	9.51065(6)	9.5107(3)
	volume / $Å^3$	136.264(1)	136.264(1)	136.265(1)
	composition / %wt.	67.55(5)	67.00(5)	66.36(6)
	oxygen coordinate, O_z	0.20767(6)	0.20770(6)	0.20746(6)
	Ti beq / $Å^2$	0.414(6)	0.416(6)	0.420(6)
	O beq / $Å^2$	0.52(1)	0.50(1)	0.509(1)
TiN/TiNO	space group		$Fm\bar{3}m$	
	lpa / Å	4.184230(3)	4.184230(3)	4.1843(1)
	volume / $Å^3$	73.260(1)	73.260(1)	73.262(1)
	composition / %wt	32.45(5)	33.90(5)	33.64(6)
	Ti coordinates		(0, 0, 0)	
	Ti occ.	0.9998(8)	0.9021(7)	0.9507(8)
	Ti beq / $Å^2$	0.76(1)	0.77(1)	0.80(1)
	N coordinates		(0, 0, 0.5)	
	N occ.	1.476(4)	1.010(3)	1.076(3)
	N beq / $Å^2$	0.87(2)	0.50(2)	0.56(2)
	O coordinates		(0.25, 0.25, 0,25)	
	O occ.			0.030(3)
	O beq / $Å^2$			0.0(4)

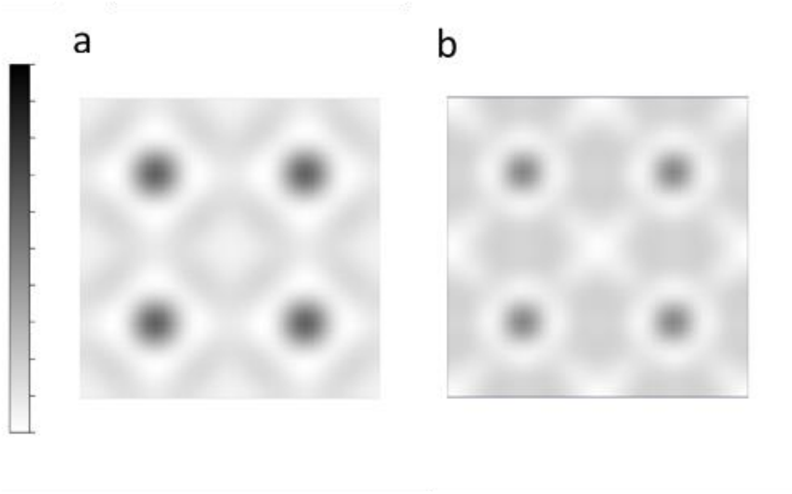

Figure S13. Fourier difference electron density map from using: (a) a standard titanium nitride phase, (b) a titanium oxynitride phase. The colour scales are the same for both maps. Plane shown is at 0.25×*a* along (001).

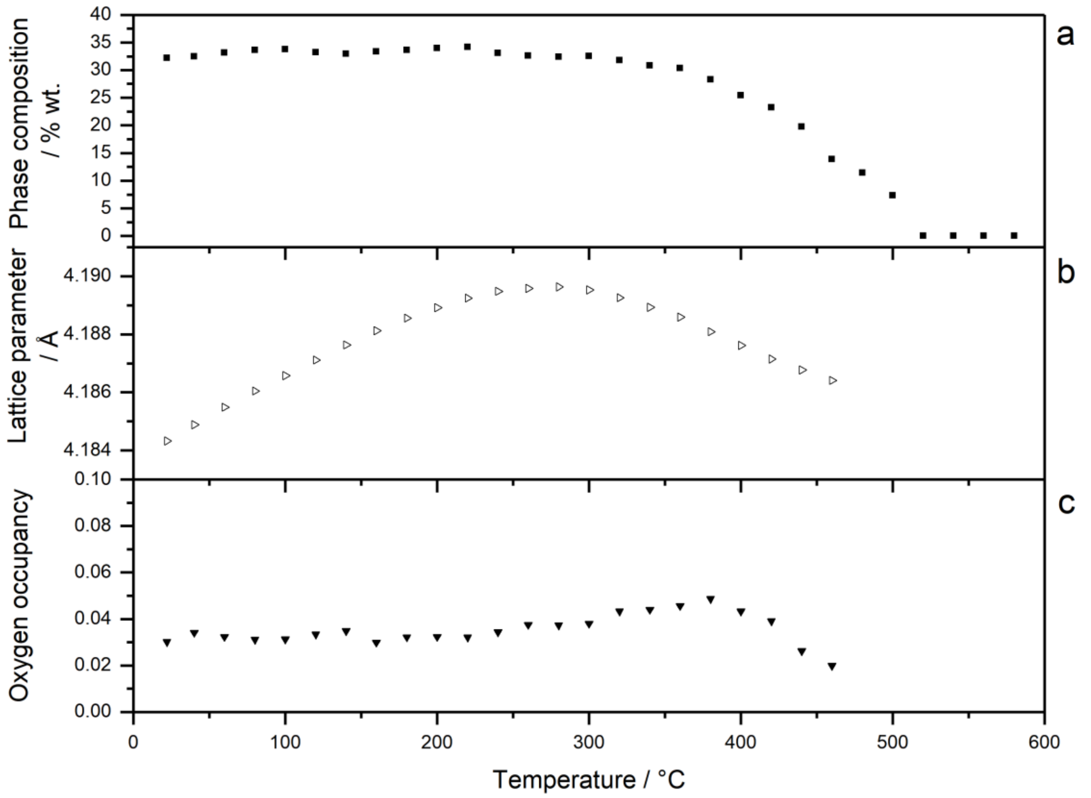

Figure S14. Structural parameters of $TiNO_x$ from the Rietveld refinement of A-750 during thermal oxidation; (a) phase composition, (b) lattice parameter *a*, (c) occupancy of interstitial oxygen site.

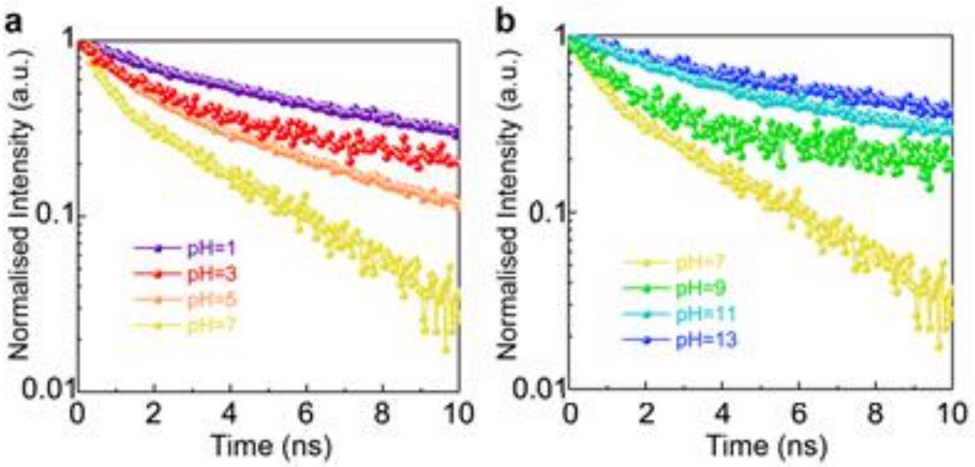

Figure S15. Time-resolved photoluminescence (TRPL) measurements of N-doped TiO_2 photocatalysts under different conditions. **(a)** P3 after being soaked in acidic solutions with different pH; **(b)** P3 after being soaked in alkaline solutions with different pH.

Temperature Effect and Uniqueness of N-doped TiO_2

This is the paper we address on the structural changes of N-doped TiO_2 for water splitting and mechanism elucidation followed by our earlier publication in the same journal mainly on the temperature effect and catalyst optimization in water splitting over the N-doped TiO_2 [4] and our recent invited review on potential advantages of photocatalysis at elevated temperatures [6].
In brief, we previous found that the hydrogen production activity over N-doped TiO_2 is 6746 μmol $g^{-1}h^{-1}$ at 270°C, which is substantially higher than the same catalyst and also most other photocatalytic systems at room temperature claimed in the literature. We postulated in these papers that at elevated temperature, the improvement in kinetics could be one of the main reasons to overcome the rate limiting regeneration of lattice oxygen vacancies from this system.
In N-doped TiO_2, we noted an anisotropic thermal expansion of anatase at 150°C, namely that the c-axis exhibits increased thermal expansion while that of the a-axis decreases. This has been attributed to the increased weakening of the long Ti-Oax bond. At 200°C the phenomenon is countered which is attributed to the Jahn-Teller contraction of 5-fold coordinated Ti^{3+} polyhedra formed by the onset formation of oxygen vacancies in sub surface (such diffraction method cannot measure the surface oxygen vacancies directly).
This onset of sub surf ace oxygen-vacancy formation in the asymmetric nitrogen-doped anatase phase at 200°C also correlates with our onset in activity as shown in Figure 5b. The activation barrier for hydrogen evolution from photocatalytic water splitting is determined to be 60.6 kJ mol^{-1}, which is in good agreement with the activation barrier for oxygen-vacancy formation of 62.5 kJ mol^{-1}, which we derived from variable-temperature EPR (Figure S8 and S9). Additionally, this formation energy is in good agreement with the reported activation energy for oxygen-vacancy mobility in anatase of 67.5 kJ mol^{-1} (see ref.11 of main manuscript) which indicates that the formation of oxygen vacancies in anatase is limited by the diffusion of vacancies between the sub-surface and the surface.

For potential practical applications, in those cited papers, we mentioned that they are reported configurations and prototypes of solar concentrators, such as parabolic cylinder reflectors[7] that can provide enhanced light irradiation as well as temperature for small/medium size applications. In addition, we have also highlighted in the papers that some strategies for example, the recovery of heat from superheat steam and using a number of possible exothermic coupling reactions with H_2 to provide the heat required for the system at large scale, etc. There are also devised exploitation plans to address some practical issues for potential applications including the injection of separated H_2 from photo splitting at elevated temperature for decentralized domestic devices into natural gas pipeline in UK and some parts of Europe, etc for caloric use of this renewable fuel.
Besides, further preliminary study has also been initiated by replacing the liquid water with water vapour, which could be more controllable, easier to operate, possess lower heat capacity (therefore uses less energy to heat up), and can be operated at lower pressure for the same temperature, etc. In another word, substituting liquid water with water vapour in continuous flow could make this system more practical and feasible at elevated temperature. It is noteworthy that the visible-light-driven water splitting system clearly works well even with water vapour, and lower pressures of water vapour have been briefly studied (in SI ref. [4]). We therefore hope that this structural study if published could stimulate further works to address some practical issues in photocatalytic splitting.
Notice that it is unclear whether surface or lattice oxygen vacancy plays the key role in catalysis at the elevated temperature in the literature. But, in this paper, the in-depth structural study of rutile, anatase and mixed phases (P25 particle) with and without nitrogen-doping by variable-temperature synchrotron X-ray powder diffraction (VT-SXPD) give for the first time that the good correlations of the anisotropic thermal expansion in lattice parameters (influenced by sub-surface oxygen vacancy), nitrogen-doping level and photocatalytic activity with respect to temperature. At high level of nitrogen-doping of anatase, a new cubic titanium oxynitride phase is also identified, which gives important hints on the fundamental shift in absorption wavelength, leading to excellent photocatalysis in visible light regime.

Although pure TiO_2 and N-TiO_2 show similar temperature dependence, since the favourable formation sub-surface oxygen vacancies over TiO_2 based material is anticipated. (supported by the calculations). But for nitrogen-doping in N-TiO_2, the inclusion of nitrogen atoms, either as an interstitial or substitutional dopant, can apparently increase the concentration of the sub surface oxygen vacancies and Ti^{3+}, and also narrow the band gap resulting in photocatalytic water splitting by visible light. This can demonstrate the unique roles of N doping in the N-TiO_2 for the photocatalytic water splitting at elevated temperature.

References

1. Ito, Y. & Goto, T. Electrochemistry of nitrogen and nitrides in molten salts. *J. Nucl. Mater.* **344**, 128–135 (2005).
2. NIST Standard Reference Database 121: Values of the Fundamental Physical Constants.
3. Arblaster, J. W. Crystallographic Properties of Platinum. *Platin. Met. Rev* **41**, 12 (1997).
4. Li, Y. *et al.* Photocatalytic water splitting by N-TiO 2 on MgO (111) with exceptional quantum efficiencies at elevated temperatures. *Nat. Commun.* **10**, 4421 (2019).
5. Zhao, W.-N. & Liu, Z.-P. Mechanism and active site of photocatalytic water splitting on titania in aqueous surroundings. *Chem. Sci.* **5**, 2256–2264 (2014).
6. Li, Y. & Tsang, S. C. E. Recent progress and strategies for enhancing photocatalytic water splitting. *Mater. Today Sustain.* **9**, 100032 (2020).
7. Pinaud, B. A. *et al.* Technical and economic feasibility of centralized facilities for solar hydrogen production via photocatalysis and photoelectrochemistry. *Energy Environ. Sci.* **6**, 1983–2002 (2013).
